# A case of propafenone toxicity in the setting of dehydration and acute kidney injury

**DOI:** 10.21542/gcsp.2024.38

**Published:** 2024-08-01

**Authors:** Enad Haddad, Matthew Collins

**Affiliations:** 1 Department of Internal Medicine, Jefferson Abington Hospital, Abington, PA, USA; 2 Department of Cardiology, Jefferson Abington Hospital, Abington, PA, USA

## Abstract

Propafenone is a class 1C antiarrhythmic and is one of the first-line drugs used in the management of atrial fibrillation. Its toxicity is rare, yet potentially life-threatening. Common clinical findings could range from hypotension, dysrhythmias, and conduction disturbances to cardiac arrest. We present a case of an 80-year-old male who presented with generalized weakness and polyuria secondary to over-diuresis leading to dehydration. Electrocardiogram on admission showed a first-degree atrioventricular block, QRS complex widening, and QTC interval prolongation. These findings were attributed to propafenone toxicity in the setting of dehydration and increased serum propafenone concentration. In the case described the optimization of fluid status and holding propafenone temporarily led to rapid reversal of the electrocardiogram changes. Multiple treatment modalities have been attempted, but standard recommendations for propafenone toxicity management have yet to be established. This case stresses the importance of taking into consideration volume status and other reversible risk factors possibly contributing to propafenone toxicity.

## Introduction

Propafenone is a class 1C antiarrhythmic and one of the first-line agents in the management of supraventricular tachycardias, particularly atrial fibrillation. While uncommon, several case reports have documented an array of cardiac and extracardiac adverse reactions to propafenone toxicity. It could be produced by the recent commencement of the drug, intentional overdose, drug-drug interactions, et cetera. While several treatment approaches have been successful, guidelines for propafenone toxicity management are yet to be established. We present a case of acute propafenone toxicity in the setting of dehydration and acute kidney injury.

### Case presentation

An 80-year-old male with a past medical history of paroxysmal atrial fibrillation on Eliquis and propafenone (425 mg twice daily), hyperlipidemia, hypertension, coronary artery disease, and stage 3A chronic kidney disease, presented to the emergency room (ER) with generalized weakness and polyuria which started 2–3 weeks ago.

His symptoms started as on-and-off weakness, especially in his legs, noticed during physical therapy and on exertion which would resolve at rest. He also felt thirsty, dry, and urinated more frequently. Two weeks prior, he experienced bilateral ankle swelling, leading to a change in diuretic dose from furosemide 20mg daily to torsemide 10 mg daily, accompanied by fluid restriction.

**Figure 1.**
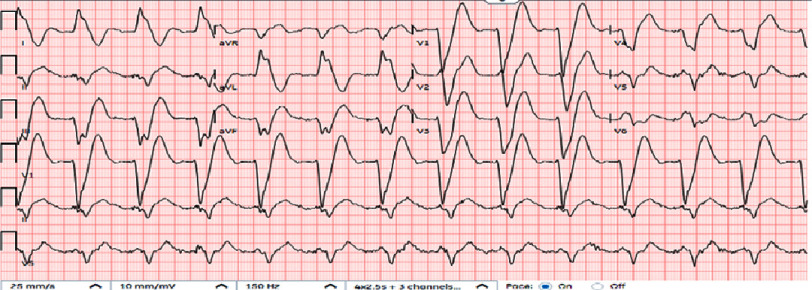
Electrocardiogram (EKG) showing sinus rhythm with a first-degree AV block, left bundle branch block, prolonged QTC interval, and a significantly widened QRS interval, without ST elevation.

In the ER, the patient was hemodynamically stable (Temp 96.7°F, heart rate 69 bpm, blood pressure 158/67 mmHg, respiratory rate 16 bpm, oxygen saturation 99% on room air). Electrocardiogram (EKG) showed sinus rhythm with a first-degree AV block, left bundle branch block, prolonged QTC interval, and a significantly widened QRS interval, without ST elevation (Figure 1). The patient’s EKG a month before showed a QTc interval of 490 ms and a QRS interval of 80 ms.

Lab results on admission were as follows: creatinine 1.80 (baseline 1.5, normal 0.7–1.4 mg/dL), blood urea nitrogen 36 (normal 12–27 mg/dL), Hemoglobin 13.4 (normal 14.0–17.0 g/dL), Troponin T peaked at 27 (normal <19 ng/L), and thyroid stimulating hormone was 1.34 (normal 0.3–5.0 uIU/mL). Albumin was normal at 4.3 (normal 3.2–4.9 g/dL). Other lab work was not significant.

The patient was admitted as a case of generalized weakness and prerenal azotemia likely related to over-diuresis and dehydration. Home medications including warfarin, carvedilol, losartan, and amlodipine, were continued. Sertraline was held due to QTc prolongation.

On admission, the patient was evaluated by Nephrology, who recommended holding diuresis and continuing gentle fluid resuscitation. Cardiology evaluation suggested EKG changes consistent with antiarrhythmic drug toxicity, likely due to volume contraction and increased drug concentration. Propafenone was temporarily held. Within 24 h, the patient’s symptoms and volume status improved, and he was able to ambulate independently.

**Figure 2.**
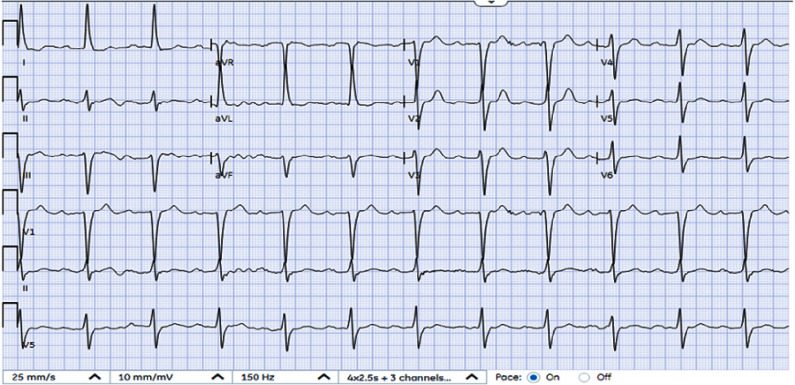
After withholding propafenone, patient showed normalized QRS interval, and the QTc interval decreased to 465 ms.

EKG the next day showed a normalized QRS interval, and the QTc interval decreased to 465 ms (Figure 2). Renal function gradually improved, and creatinine normalized to 1.37. On day 2 post-admission, Cardiology recommended restarting propafenone at 150 mg every 8 h along with continued cardiac monitoring and daily EKGs. Telemetry and daily EKGs over the next 2 days revealed no clinical arrhythmia, and QRS and QTc intervals were maintained at baseline. On day 6 post-admission, the patient returned to his baseline health status and was discharged on a lower dose of propafenone of 325 mg twice daily.

## Discussion

We present a case of a patient who is on long-term propafenone treatment for paroxysmal atrial fibrillation. He was on a high dose of rythmol (425 mg twice daily) as this dose maintained him in sinus rhythm most of the time, especially after multiple antiarrhythmics were ineffective or produced intolerable side effects.

He presented with weakness secondary to dehydration and was incidentally found to have abnormal EKG changes which were attributed to propafenone toxicity in the setting of volume contraction and increased propafenone serum concentration. The patient had not taken any extra doses, nor had he started any medications that would interact with propafenone in the recent period before hospital presentation. His albumin level was normal and other lab workup did not show electrolyte or cardiac enzyme abnormalities, and neither did the patient have other arrhythmias. In light of the aforementioned details, the patient’s EKG changes were most likely attributed to propafenone toxicity in the setting of excessive diuresis and consequent propafenone accumulation.

### Propafenone

Propafenone is a sodium channel blocker and belongs to class 1C as per the Vaughan-Williams classification. It also has beta-adrenergic blocking activity, especially in its non-metabolized form^1^. Propafenone is one of the first line agents recommended for the pharmacological conversion of atrial fibrillation (commonly referred to as “pill-in-the-pocket” treatment) and is used for the maintenance of normal sinus rhythm in atrial fibrillation patients without a significant underlying structural heart disease (including patients with heart failure and decreased left ventricular function)^2,3^. Propafenone is metabolized in the liver *via* the CYP450 2D6 pathway. In patients with intact CYP450 2D6 metabolism, the half life is between 2–12 h, with a mean of 6 h^4^. Slow metabolizers and patients who consume drugs that are known to inhibit the P450 enzymes are at higher risk of propafenone toxicity, particularly the beta-adrenergic blockade effects^1^.

### Propafenone toxicity

Propafenone toxicity manifests in a variety of cardiac and extracardiac effects. The risk of cardiovascular adverse effects was shown to be 5% in large studies. These include conduction disturbances (sinus bradycardia, sinoatrial block, sinus arrest, atrioventricular block, and bundle branch blocks), induction of dysrhythmias (ventricular tachycardia, atrial and ventricular flutter, atrial fibrillation), transient hypotension, and reversible widening of the QRS complex. Extracardiac side effects include nausea, dizziness, gastrointestinal disturbances, and headaches^5^.

Diagnosing propafenone toxicity should include taking careful history including a recent initiation of propafenone or an increase in its dose, and recent intake of CYP450 inhibiting drugs, including alcohol. Common EKG changes seen with propafenone toxicity include prolongation of the PR interval, bundle branch blocks, widening of the QRS and the QT intervals, bradycardia, and ventricular tachycardia^4^. Measuring serum propafenone levels has been used in a few instances to confirm the diagnosis but there is little information on its utilization in the management of propafenone toxicity. Nevertheless, serum propafenone levels were shown to correlate with the degree of QRS widening^6^.

### Treatment of propafenone toxicity

There are currently no standard guidelines or an established antidote for propafenone toxicity treatment. However, multiple approaches have been implemented which were shown to be effective. The initial management should include immediately discontinuing the medication and ruling out concurrent use of possible cyp450 inhibitors which could slow the metabolism of propafenone^1^. Supportive care should include continuous cardiac monitoring and optimizing fluid status and blood pressure^6^. Sodium bicarbonate has been successfully used in propafenone toxicity where it was shown to reduce the QRS duration and improve hemodynamic status within hours of treatment. Additionally, in cases where propafenone toxicity manifested as symptomatic bradycardia, cardiac pacing has proven to be effective^6,7^.

In our patient, prompt fluid resuscitation and propafenone cessation were effective in reversing the initial EKG abnormalities seen upon presentation. This indicates that the EKG changes observed in our patient were most likely a consequence of an elevated serum concentration of propafenone. The patient’s EKG changes were resolved within 11 h of the first EKG, which is about 1–2 half-lives of propafenone, assuming that the patient is not a slow metabolizer. However, his metabolizer status was not known. Ultimately, restarting propafenone at a lower dose after the resolution of AKI and restoring fluid status did not elicit a recurrence of the EKG changes, as confirmed by serial EKGs.

### What have we learned?

• In patients exhibiting EKG changes on propafenone therapy, factors such as dehydration and impaired drug metabolism (including slow metabolizers and concomitant use of cytochrome P450 inhibitors) should be considered during the evaluation of the case.
• Optimizing volume status and reversing kidney injury are essential factors to consider when managing propafenone toxicity.

## Funding

This research has not received any specific grant from public, commercial, or non-profit sector agencies.

## Conflicts of interest

The authors have no conflicts of interest to declare

## Author Contributions

**Conceptualization**: Enad Haddad and Matthew Collins. **Writing** - original draft: Enad Haddad. **Writing** - review & editing: Enad Haddad and Matthew Collins.
